# Central Dental Association of Northern New Jersey

**Published:** 1892-03

**Authors:** 


### CENTRAL DENTAL ASSOCIATION OF NORTHERN NEW JERSEY.

Regular meeting held Monday evening, April 20, 1891, at Newark, N. J. The President, C. W. F. Holbrook, in the chair.

Richardson Grey, M.D., of East Orange, read a paper entitled "We Specialists."

(For Dr. Grey's paper, see page 177.)

*The President.*—Gentlemen, the paper is before you for discussion. Dr. Bogue, may we hear from you?

*Dr. Bogue.*—I should like to make my thanks to the essayist. He has brought forward a point or two that seems to me unusual in precedent.

We are as a profession crushed; the State has taken hold of us and excluded me from practice in it. While not complaining of that, either, I was not a little surprised to find that the doctor had noticed the exclusion of the word State. It has come to me, also, that this is a specialty of a specialty. Gas is not the only specialty we practise.

Dr. Grey, in referring to our exclusion from the medical profession, has not taken into account an item of history, that forty-five or fifty years ago we, I mean the dentists, asked for a chair in a medical college, in order that students might study medicine; might get a ground-work of anatomy, physiology, pathology, etc., etc. We were refused, and it was only after the Baltimore College was organized that we were granted this privilege. From that time we have extended our knowledge and we have been known as specialists within and without general medicine. But very much as our country began with a protective tariff, and is coming gradually to free trade, so we dentists began as tooth-pullers, and have worked our way up until we were received at Washington at the last Medical Congress, and our profession as a specialty of medicine recognized, and we were given a stimulus that we have felt ever since.

Another stimulus was offered a few years ago by which only graduates of two of our American colleges were recognized in England. A great hue and cry was made both here and there, and the result was that our standard of dental education was raised, and from that date onward we must answer for three years in our dental schools; a very creditable condition of things, largely due, it seems to me, to that law which for a time appeared to us most oppressive. I venture to speak on these matters, as they have been extensively touched. I want to give the doctor a few words of encouragement in the fact that we are striving towards the point which he has so equivocally given us.

*Dr. Domingo M. Salater.*—Many diseases of the throat and ear are connected with the practice of dentistry. I saw a case of pharyngitis from the improper development of the wisdom teeth, and the patient was in the hands of an M.D. for fourteen years. Finally he went to a dentist, and when those teeth were brought out nicely and everything was clear, his condition was better, and he was cured within three weeks.

I will hope that a future time may come when the relations between the M.D. and the D.D.S. will be more intimate, and I think that we will then accomplish a great deal more for each other.

*Dr. Meeker.*—In reference to the advertisement of "fresh gas daily," I want to say that every dentist here knows that fresh gas is not good gas, that the anæsthetic properties of gas that has been purified are much better for the public use than gas that is fresh.

*Dr. Mills.*—Mr. President, I do not know that I can say a great deal on this subject. I agree with Dr. Bogue, and want to make my thanks to the essayist.

I regard the reading of medical papers before us as very timely. I certainly think we have a great deal to learn from the medical profession, and I think, too, that medical men have much to learn from us, dentists.

We should be sympathetic workers for progress, and we certainly should accept suggestions from our medical friends graciously.

*Dr. Stockton.*—I want to say that the essayist has brought out some points that are well worthy of our consideration. While we are not educated medically, yet I want to take the ground that we are medically valuable; the teeth holding a high place in the human organism, quite as important as any other organs, and as a necessary sequence we should be as educated as are medical men. I say to-day, as I have often said before, that the man who expects to be a successful practitioner of dentistry should know the whole system from the top of the head to the sole of the foot. You cannot stop just at the teeth; dentistry is not a mere matter of being able to extract a tooth or put in a plug.

I want to emphasize this point, that according to the provisions of the State law a man who is not qualified cannot come into practice. Dr. Bogue very well said that we are now to-day recognized as part of the medical profession.

*Dr. Bogue.*—Mr. President and gentlemen, may I be allowed a moment? I want to announce what may, perhaps, seem a heresy, after Dr. Stockton's remarks and those of the gentleman *en face.* I know so little about gas that some of the points brought up this evening are new to me. I want to proclaim that I do not know enough to extract a tooth that is not pulled out by a string, except in a few cases where the deformity is so great that no other course could be taken. It seems to me that we might possibly be justified in saying to the student, "Whenever you extract a tooth under such a circumstance, necessarily the extraction of a tooth is the acknowledgment of incompetency." Possibly our knowledge reaches its limit in such cases. There are conditions beyond which we know nothing, and so we give it up.

*Dr. Osmun.*—The discussion has taken quite a wide range. To the last remark of Dr. Bogue I would take exception. He says that the extraction of a tooth is the admission of incompetency. I cannot believe that, and there are times when extraction is admirable surgery, and must be resorted to. I have seen some cases in my short practice when it was good surgery, and was not an acknowledgment of incompetency.

The administration of anæsthetics in dentistry is like the administration of anæsthetics anywhere else. With careful supervision it is right, proper, and to be commended. The first object of a dentist is to relieve pain, and the right use of anæsthesia is an important adjunct in this connection.

The question of the education of the public is a very commendable one. The educated patient is always the appreciative patient, and every professional man ought to be a teacher. It is his right, his privilege, and his duty to educate every patient who comes into his chair.

### REPORT OF SPECIAL COMMITTEE OF FIVE RELATIVE, TO RESOLUTIONS ON THE DEATH OF DR. ATKINSON, M.D., D.D.S.

*Dr. Luckey.*—Mr. President and gentlemen, you will, of course, recollect that when the news of Dr. Atkinson's death reached us it was agreed that certain resolutions should be passed, and a committee of five were appointed for that purpose, consisting of Drs. Osmun, Stockton, Meeker, Watkins, and Holbrook, and it was suggested that we draft a series of resolutions for your endorsement.

*Resolved*, That a Memorial Committee be established or instituted, composed of two members each from the First District Dental Society of New York, the Odontological Society of New York, Second District Society of Brooklyn, Central Dental Association of Northern New Jersey, and the New Jersey State Dental Society, making a committee of ten, who shall be empowered to organize themselves, and call upon the whole world for a generous response, to the end that a suitable memorial be erected to the memory of our lamented friend Dr. Wm. H. Atkinson.

B. F. Luckey.

Remarks were made on the life and character of Dr. Atkinson by Drs. Stockton, Luckey, Watkins, and Levy, after which the resolution was passed.

Adjourned.

